# Promising therapeutic mechanism for Chinese herbal medicine in ameliorating renal fibrosis in diabetic nephropathy

**DOI:** 10.3389/fendo.2023.932649

**Published:** 2023-07-14

**Authors:** Shengju Wang, Shuai Qin, Baochao Cai, Jihong Zhan, Qiu Chen

**Affiliations:** ^1^ Department of Nephrology, The First Affiliated Hospital of Guizhou University of Traditional Chinese Medicine, Guiyang, Guizhou, China; ^2^ Department of Endocrinology, Hospital of Chengdu University of Traditional Chinese Medicine, Chengdu, Sichuan, China; ^3^ Diabetes Department, Jiaxing Hospital of Traditional Chinese Medicine, Jiaxing, Zhejiang, China

**Keywords:** diabetic nephropathy, renal fibrosis, Chinese herbal medicine, oxidative stress, ASK1

## Abstract

Diabetic nephropathy (DN) is one of the most serious chronic microvascular abnormalities of diabetes mellitus and the major cause of uremia. Accumulating evidence has confirmed that fibrosis is a significant pathological feature that contributes to the development of chronic kidney disease in DN. However, the exact mechanism of renal fibrosis in DN is still unclear, which greatly hinders the treatment of DN. Chinese herbal medicine (CHM) has shown efficacy and safety in ameliorating inflammation and albuminuria in diabetic patients. In this review, we outline the underlying mechanisms of renal fibrosis in DN, including oxidative stress (OS) generation and OS-elicited ASK1-p38/JNK activation. Also, we briefly summarize the current status of CHM treating DN by improving renal fibrosis. The treatment of DN by inhibiting ASK1 activation to alleviate renal fibrosis in DN with CHM will promote the discovery of novel therapeutic targets for DN and provide a beneficial therapeutic method for DN.

## Introduction

Approximately 50% of diabetes patients develop diabetic nephropathy (DN) (1). DN is one of the major microvascular diseases of diabetes mellitus and is currently the most common cause of chronic renal failure among diabetic patients (2–5). DN has significant long-term impacts on the morbidity and mortality of diabetes patients. The distinctive features of DN are glomerular basement membrane thickening, matrix accumulation in both mesangium and tubulointerstitium, and podocyte dysfunction or depletion, leading to the occurrence of renal function decline (6–9).

Fibrosis is a process marked by excessive extracellular matrix (ECM) deposits that contribute to functional parenchyma damage by fibrotic tissue (10). The role of fibrotic alterations in DN development has been identified as a critical in favoring chronic renal failure in diabetes mellitus (11, 12). Studies have confirmed that the grade of the fibrotic changes within the renal cortical interstitium is positively correlated with serum creatinine levels in adults with DN (13–15). Renal fibrosis is characterized by certain pathological hallmarks encompassing myofibroblast activation, ECM, and renal inflammation. The kidney gradually becomes incapable of repairing itself because of progressing tissue lesions and inflammation, eventually resulting in renal fibrosis (16–18). However, renal fibrosis is a multifactorial and dynamic process that induces many cellular changes in response to injurious triggers. Studies have recognized many factors involved in the development of fibrosis of DN, including oxidative stress (OS), transforming growth factor (TGF)-β, and inflammatory cytokines (such as IL-1/6 and TNF-α) (19, 20).

## OS and renal fibrosis

OS is always followed by an increase in the production of reactive oxygen species (ROS). A continuous hyperglycemic state contributes to the occurrence of OS when the local antioxidant functionality loses its ability to quench the overproduction of ROS (21, 22). Sufficient evidence has confirmed that OS has a significant impact on the pathogenesis of multiple diabetic complications, such as DN (23). OS is increased in diabetic renal tissue due to elevated inflammation, hypoxia, overproduction of NADPH oxidases, exhaustion of antioxidant proteins, and mitochondrial dysfunction (24). OS is one of the principal factors that hasten glomerulosclerosis and tubulointerstitial fibrosis, which is the common process contributing to uremia in DN (25–27). OS-activated redox-sensitive signals facilitate parenchymal cell dysfunction through apoptosis and necrosis and boost unchaining of inflammatory mediators (e.g., IL1/6 and INF-α). Inflammation and apoptosis significantly contribute to the activation and accumulation of myofibroblasts, and the overproduction of ECM cells leading to fibrosis in DN (28).

Furthermore, sustained hyperglycemia and OS promote the excessive production of advanced glycation end products (AGEs) (29). When the AGEs that are primarily cleared by the kidneys are elevated (30), they can induce the glomerular cells to release transforming growth factor-β1 (TGF-β1), causing glomerular sclerosis and interstitial tubular damage in response to the elevated production of ECM (31, 32). The augmentation of ECM results in renal fibrosis mainly produced by the recruitment of mesangial cells, promoting ECM accumulation, thickening of the glomerular and tubular membranes, and disability of the podocytes, finally causing cell death (33, 34).

## Apoptosis in renal fibrosis

Apoptosis is characterized by a decrease in volume, cell surface blebbing, chromatin shrinkage, internucleosomal fragmentation of DNA, and production of apoptosis bodies; all these processes are indispensable in the maintenance of normal tissue homeostasis (35). However, excessive apoptosis or its dysfunction causes various pathological fibrosis processes (36). It has been shown that a persistent hyperglycemic state elicits apoptosis and leads to the decline of renal function in DN (37). A hyperglycemic environment amplifies apoptosis in multiple cell types within DN, encompassing the proximal tubule epithelial, endothelial, and interstitial cells (38, 39). Renal interstitial fibrosis is the ultimate common pathological consequence of chronic renal disease (40, 41), marked by tubular atrophy and ECM deposition (42). Apoptosis of the tubular epithelial cells is one of the hallmarks of tubular atrophy and interstitial fibrosis (43, 44). Apoptosis has been found in renal tubular cells from patients with DN, while the blockade of renal tubular cell apoptosis retards fibrosis (45–47).

OS and ROS are identified as apoptosis stimulators and regulators (48, 49). Exogenous ROS causes apoptosis in many types of cells (50); however, OS and ROS alone are incapable of inducing apoptosis, thus requiring the involvement of other cell death signaling pathways.

## ASK1 and p38/JNK

### p38/JNK and renal fibrosis

Renal biopsy analysis from patients has displayed overexpression of p38 in many glomerular injuries and DN (51, 52), especially within proliferative forms of glomerulonephritis (51). Overproduction of p38 promotes the accumulation of numerous proinflammatory and profibrotic cytokines, such as TNFα, TGF-β1, and monocyte chemoattractant protein-1 (MCP-1). In humans and rodents with DN, p38 is overexpressed in a wide range of renal cells, including podocytes, endothelial cells, tubular cells, mesangial cells, macrophages, and myofibroblasts, and is associated with disease development (51–53). These studies indicate that p38 signaling is a great contributor to the development of kidney inflammation and fibrosis.

There is accumulating evidence confirming the notion that c-Jun N-terminal kinase (JNK) serves as a powerful proapoptotic mechanism in stressed cells (54). It has been found that overexpression of JNK is capable of driving the production of apoptogenic mediators such as cytochrome *c* (55), as well as proinflammatory and profibrotic mediators such as IL-6 and TNF-β1 (56, 57), which contribute to fibrotic renal disease (58). Overactivation of JNK in renal cells can favor fibroblast and collagen production. Simultaneously, JNK signaling can hasten profibrotic TGF-β1 signaling, playing an essential role in the progression of fibrotic kidney disease (59). JNK expression activated by mediators such as TNF-α, IL-1/6, and angiotensin II promotes TGF-β1 generation via stimulation of the activator protein-1 (AP-1) (60, 61). Additionally, TGF-β1 positively promotes mitogen-activated protein kinase (MAPK) activation through ASK1 (62, 63). The JNK signal transduction pathway also promotes the tubular generation of thrombospondin-1, leading to positive feedback that stimulates the production of TGF-β1 (61). Hence, overexpression of JNK in renal cells, especially in tubular epithelial cells, causes ongoing renal injuries with renal cell necrosis followed by apoptosis leading to tubulointerstitial lesions via the enhancement of inflammation and fibrosis (64–67).

### ASK1

Numerous publications indicate that novel apoptotic pathways play a vital role in the pathogenesis and progression of DN. Apoptosis signal-regulating kinase (ASK) 1 is an upstream kinase in the MAPK pathway. The MAPK pathway promotes OS-elicited renal apoptosis, kidney inflammation, and fibrosis, favoring DN development (68–70). Increased expression levels of ASK1 have been identified in kidney biopsy tissues from adults with DN (71). ASK1 stimulates the activation of p38 and JNK in response to the pathological OS, modulating different stress-induced responses in development and cellular function (72). The downstream MAPKs, p38, and JNK are significantly elevated in a range of renal tissues such as the glomerulus, vasculature, and renal tubule interstitium, thereby inducing apoptosis, inflammation, fibrosis, and renal damage in humans (73–75).

### ASK1-p38/JNK pathway

Studies have shown that the deposition of glomerular ECMs is elevated as long as the MAPK signaling pathways are activated in the diabetic state. ROS quickly stimulates p38 and JNK through distinct signaling pathways, such as ASK1. Additionally, inhibitors of p38/JNK by either genetic or pharmacological methods enhance the inhibition of apoptosis activated by either ASK1 or ROS. Experiments have shown that in ASK1^−/−^ cells, p38/JNK is not stimulated in response to ROS. Decreased expression levels of p38/JNK are critical underlying mechanisms of resistance to ROS-induced apoptosis (76). Activation of a preponderant negative mutant of p38/JNK also improved resistance to ROS-elicited apoptotic cell death (77). Hence, inhibition of the ASK1-p38/JNK pathway can be an important approach that protects against OS-induced apoptosis, which is a central mechanism of renal inflammation and fibrosis in DN (78–80).

## Therapeutic strategies for renal fibrosis

### Antioxidants

It is evident that oxidative injury induces apoptosis, necrosis, and cellular dysfunction in many pathologies (81, 82), implying that antioxidants may be an effective treatment for many diseases (82, 83). Although many ROS inhibitors have been recommended to treat DN, they fail to scavenge ROS within DN due to non-specificity, impotence, and insufficient pharmaceutical activity (84, 85). Moreover, the actions of the antioxidants may be based on interference within ROS-induced pathways (84, 85). Hence, effective antioxidant therapies should be focused on an understood specific OS/ROS-related target or pathway that contributes to the pathological process of a disease in a defined organ or tissue. Specific targets have been proposed that will benefit the pathological progression of oxidative damage while avoiding direct targeting of non-specific OS or ROS. Therefore, the ASK1-p38/JNK axis is a specific mechanism driving the progression of myofibroblast generation and renal inflammation and fibrosis in DN, making it a beneficial target for treating DN.

### p38 or JNK inhibitor

Selective inhibition of p38 is efficacious in rodents with renal damage (51, 86, 87). However, these outcomes have not been replicated in clinical practice due to the impotence and side effects on other organs and tissues (88). Hence, the application prospect of p38 inhibition in a clinical setting is still uncertain. Clinical trials have demonstrated that JNK inhibitors alleviate fibrosis in lung and liver disease (89, 90). Studies have also shown that JNK inhibitors can block the progression of fibrotic renal changes in a rodent model (91, 92). However, many questions exist, primarily about the side effects. Studies have shown that although JNK inhibition in rodents can significantly protect the kidneys from inflammation and fibrosis damage, it can also aggravate albuminuria while alleviating renal inflammation (93–96). Moreover, the relative action of JNK1 and JNK2 in different types of renal damage is still unestablished. Furthermore, whether the combined JNK1 and JNK2 inhibitions will impact normal health is still unknown. Also, the application of JNK inhibition is not well understood, considering the actions of JNK expression in a wide range of cells following renal damage. p38 and JNK inhibitors have exhibited both helpful and detrimental actions within disease states (91); thus, there may be limitations to inhibiting all p38 and JNK targets as a disease state therapeutic. Therefore, the upstream kinases of the p38/JNK pathway could be an alternative target for fibrotic renal disease in DN.

### ASK1 inhibition

ASK1 is a critical mediator of apoptosis, inflammation, and renal fibrosis and is an upstream kinase in the p38 and JNK pathways (95). ASK1 inhibition in clinical trials has already shown promising antifibrotic efficacies in non-alcoholic steatohepatitis (NASH) (97). Therefore, it will be beneficial to clarify whether the actions are translated into fibrosis within DN. Inhibition of ASK1 can halt inflammation, myofibroblast production, and collagen accumulation in rodents with fibrotic kidney disease (98). Data demonstrate that ASK1^−/−^ mice develop normally. However, they have delayed pathological organ remolding, fibrotic changes in multiple disease states, reduced apoptosis-induced cell loss, and ROS generation (99–101), implicating that therapeutic targeting of ASK1 can be a viable way to inhibit the progression of oxidative damage, organ remolding, and fibrotic changing (102).

### Chinese herbal medicine

Although current drugs such as angiotensin-converting enzyme inhibitor (ACEI) and angiotensin receptor blocker (ARB) are highly recommended for the treatment of DN because they can protect the renal function of people with DN via blood glucose and pressure control, they are a single-target and insufficient to delay DN progress to diabetic end-stage kidney disease (103). Therefore, discovering a beneficial agent for DN is a priority within modern medical research. Traditional Chinese medicine (TCM) is part of a therapeutic strategy widely used in treating diabetes and its complications in China for centuries due to having less toxic and adverse reactions (104–106). Herbs are the primary approach for medical treatment in TCM. Chinese herbal medicine (CHM) has shown efficacy in alleviating OS, inflammation, and albuminuria in diabetic patients (107). TCM has been increasingly used to prevent renal damage due to its clinical efficacy and safety (108). Considering the different mechanisms involved in the pathogenesis of renal fibrosis, CHM displays multiangled and multichanneled properties that are being applied in clinical practice to fight against renal fibrosis (109) (**Table 1**).

**Table 1:** Studies on the regulatory mechanism of CHM in the treatment of renal fibrosis in DN.

CHM	Study type	Targets	Outcomes	Ref.
Huangkui Capsule (HKC)	Animal study (STZ-induced male SD rats)	p38 MAPK/Akt, TGF-β1, TNF-α	Body weight ↓, kidney weight ↓, urinary albumin ↓, renal function ↑, renal fibrosis ↓	(110)
Xiexin Decoction	Animal study (db/db diabetic mice)	NF-κB, TGF-β1/Smad, ICAM-1, MCP-1, TNF-а, IL-1β, ICAM-1, collagen I/IV	Body weight ↓, right kidney weight ↓, FBG ↓, serum insulin ↓, serum TG ↓, Home-IR ↓, creatinine clearance ↑, BUN ↓, urinary albumin excretion ↓, renal fibrosis ↓	(111)
ErHuang Formula (EHF)	Animal study (high sugar-fat diet and STZ-induced male SD rats)	CXCL6/JAK/STAT3, IL-6, TNF-α, TGF-b1, collagen I/III, MMP2/9	Blood glucose ↓, weight of rats ↓, renal function ↑, renal fibrosis ↓	(112)
Acetylshikonin, the main ingredient of Zicao	Animal study (STZ-induced mice)	TGF-β1/Smad, IL-1β, IL-6, MCP-1, ICAM-1, PAI-1, CTGF	Body weight ↓, blood glucose ↓, blood pressure ↓, kidney/body weight ↓, creatinine ↓, BUN ↓, renal fibrosis ↓	(113)
Berberine (BBR)	Animal study (STZ-induced male Wistar rats)	TGF-β, α-SMA, NF− κB	Urine albumin↓, glucose ↓, creatinine ↓, BUN ↓, renal fibrosis ↓	(114)
Tongxinluo (TXL)	Animal study (KK-Ay mice *vs.* C57BL/6J mice)	TGF-β1/Smad3, collagen IV, FN	Renal function ↑, renal fibrosis ↓	(115)
Qi-dan-di-huang (QDDH) decoction	Animal study (STZ-induced male SD rats)	TGF-β1, α-SMA, IL-6, IL-1 β, TNF-α	Glucose ↓, renal function ↑, renal fibrosis ↓	(116)
Liuwei Dihuang pill (LDP)	Animal study (STZ-induced male SD rats)	TGF-β/SMADS, MAPK (p38 and ERK), NF-κB	Creatinine ↓, BUN ↓, renal function ↑, SOD and NOS ↑, MDA ↓, renal fibrosis ↓	(117)
*Coreopsis tinctoria* (AC)	Cell study (HBZY-1 cells)	TGF-β1/Smads/AMPK/NF-κB, collagen IV, FN, P-65, MCP-1	Renal inflammation and fibrosis ↓, renal function ↑	(118)
Bupleurum polysaccharides (BPs) (isolated from *Bupleurum smithii* var. *parvifolium*)	Animal study (STZ-induced C57BL/6 mice)	HMGB1-TLR4, collagen IV and FN, α-SMA, TNF-α, IL-6	Body weight ↓, blood glucose ↓, creatinine ↓, β2-MG ↓, renal swollen ↓, renal fibrosis ↓	(119)
Danggui-Shaoyao-San (DSS)	Animal study (STZ-induced male SD rats)	Jagged1, Notch1, Hes5, NICD, α-SMA, Vimentin	Renal fibrosis ↓, renal function ↑	(120)
*Taxus chinensis*	Animal study (high-fat diet and STZ-induced male SD rats)	TGF-β1/Smad	Body weight ↑, FBG ↓, renal fibrosis ↓, renal function ↑	(121)
Sanziguben Granule (SZGB)	Animal study (STZ-induced male SD rats)	Nrf2/HO-1, 4-HNE, α-SMA, Vimentin, E-cadherin, Cleaved caspase-3, Bcl-2	Blood glucose ↓, TC ↓, TG ↓, creatinine ↓, BUN ↓, GSH ↑, MDA ↓, CAT ↓, renal fibrosis ↓	(122)
*Abelmoschus esculentus* (AE)	Animal study (high-fat diet and STZ-induced male SD rats)	DPP-4, GLP-1R	Serum glucose and insulin levels ↓, HOMA-IR ↓, TG ↓, TC ↓, LDL ↓, albumin excretion ↓, urine creatinine ↓, MDA ↓, renal fibrosis ↓	(123)
Tangshen Formula (TSF)	Animal study (High-fat diet and STZ-induced male Wistar rats)	TGF-β/Smad, NF-κB, collagen I/IV, FN, MCP-1, IL-1β, TNF-α	Blood glucose →, microalbuminuria ↓, renal inflammation and fibrosis ↓, renal function ↑	(124)
Eucommia bark (Du-Zhong)	Animal study (STZ-induced male Wistar rats)	TGF-β/CTGF, Smad2/3, STAT3	Blood glucose →, BUN ↓, creatinine ↓, creatinine clearance ↑, 24-h urine volume ↓, 24-h urine protein ↓, renal fibrosis ↓	(125)
Notoginsenoside R1 (NGR1)	Animal and cell studies (db/db mice and HK-2 cells)	Nrf2/HO-1, Bcl-2, Bax, caspase-3/9, TGF-β1, collagen I	FBG ↓, TC ↓, TG ↓, 24-h urine volume and albumin ↓, β2-MG ↓, creatinine ↓, BUN ↓, renal fibrosis ↓	(126)
*Cyclocarya paliurus* (CP)	Animal and cell studies (STZ-induced male SD rats and HK-2 cells)	AMPK/mTOR, caspase-3, LC3II, P62,	Renal weight ↓, creatinine ↓, BUN ↓, renal fibrosis ↓	(127)
Oryeongsan (ORS)	Animal study (db/db mice)	TGF-β1, Smad-2/-4, collagen IV, CTGF, TIMP, Smad-7, MT1-MMP, ICAM-1, MCP-1	Body weight ↓, TC ↓, TG ↓, LDL-C ↓, blood glucose and insulin ↓, HOMA-IR ↓, creatinine clearance ↑, urine albumin ↓, BUN ↓, renal fibrosis ↓	(128)
Hu-Lu-Ba-Wan (HLBW)	Animal study (high-fat diet and STZ-induced male Wistar rats)	PKC-α/NADPH, p47^phox^, FN	Blood glucose ↓, kidney/body weight ↓, BUN ↓, creatinine ↓, urinary total protein ↓, urinary albumin ↓, TC ↓, TG ↓, LDL-C ↓, HDL-C ↑, renal fibrosis ↓	(129)
Chaihuang-Yishen granule (CHYS)	Animal study (STZ-induced male Wistar rats)	TGF-b/Smad3, collagen I/IV, FN,	Blood glucose ↓, 24-h proteinuria ↓, renal function ↑, renal fibrosis ↓	(130)
Astragaloside IV (AS-IV, *Astragalus membranaceus* (Fisch) Bge)	Animal study (STZ-induced male C57BL/6 mice)	MEK1/2-ERK1/2-RSK2, TGF-β1	24-h urinary albumin excretion ↓, UACR ↓, blood glucose and insulin ↓, TG ↓, HDL-C ↑, LDL-C ↓, renal fibrosis ↓	(131)
Chaihuang-Yishen (CHYS) granule	Animal study (STZ-induced male Wistar rats)	NF-κB p65, MCP-1, TNF-α, TGF-β1	Body weight ↓, blood glucose ↓, 24-h urinary protein ↓, TC ↓, TG ↓, BUN ↓, renal fibrosis ↓	(132)
Kangen-karyu (Guan-Yuan-Ke-Li)	Animal study (Male C57BLKS/J db/db mice *vs.* m/m mice)	RAGE, CEL, CML, GA-pyridine, TGF-β1, FN, collagen IV	Serum glucose and leptin ↓, creatinine ↓, BUN ↓, renal fibrosis ↓	(133)
Danshen	Animal study (STZ-induced male SD rats)	TGF-β1, megalin	Blood glucose ↓, AGEs ↓, LPO ↓, 24 h urinary protein excretion ↓, creatinine ↓, BUN ↓, GSH-Px ↓, SOD ↑, renal fibrosis ↓	(134)
Tangnaikang (TNK)	Cell study (HK-2 cells)	α-SMA, E-cadherin, collagen I/III, FN	Renal fibrosis ↓	(135)

SD, Sprague–Dawley; FBG, fasting blood glucose; BUN, serum urea nitrogen; TG, triglycerides; TC, total cholesterol; LDL-C, low-density lipoprotein cholesterol; HDL-C, high-density lipoprotein cholesterol; HOMA-IR, homeostasis model assessment-estimated insulin resistance; TGF-β1, transforming growth factor-β1; PAI-1, plasminogen activator inhibitor type 1; IL-1β, interleukin-1β; IL-6, interleukin-6; TNF-α, tumor necrosis factor-α; FN, fibronectin; NF-κB, nuclear factor kappa-B; LPO, lipid peroxide; SOD, superoxide dismutase; GSH-Px, glutathione peroxidase; AGEs, advanced glycation end products; UACR, urinary albumin to creatinine ratio; MCP-1, monocyte chemoattractant protein-1; NADPH, nicotinamide adenine dinucleotide phosphate; JAK/STAT, Janus kinase-signal transducer and activator of transcription; β2-MG, β2-microglobulin; ↓, reduce, decline; ↑, increase, augment.

## Conclusions and perspectives

In conclusion, modern treatments against blood glucose and pressure control can delay but inadequately block the development of multiple renal damages in DN. Hence, developing new therapeutic strategies that target the specific mechanisms of renal damage is becoming urgent. Mechanisms associated with stress-related inflammation and fibrosis are critical for kidney damage in DN and are not targeted by current small-molecule therapeutics.

The underlying pathological mechanisms of fibrosis in DN are closely associated with stress-elicited activation of the p38 and JNK pathways in various types of renal cells (91). Therapeutic inhibitors target these two protein kinases and block inflammation and fibrosis progression in animal models of renal disease (91); however, the toxic side effects such as liver toxicity, aggravation of albuminuria, and dizziness should not be ignored (88, 95, 96). Therefore, targeting the upstream signaling would be an optimal option. ASK1 is an upstream kinase of p38 and JNK and is critical for mediating numerous disease states such as apoptosis, inflammation, and renal fibrosis (95). More importantly, activation of ASK1 only happens in pathological conditions. Therefore, ASK1 can be seen as a novel target to halt the pathological progress of p38 and JNK activation in OS-related fibrotic renal change of DN.

**Figure 1:**
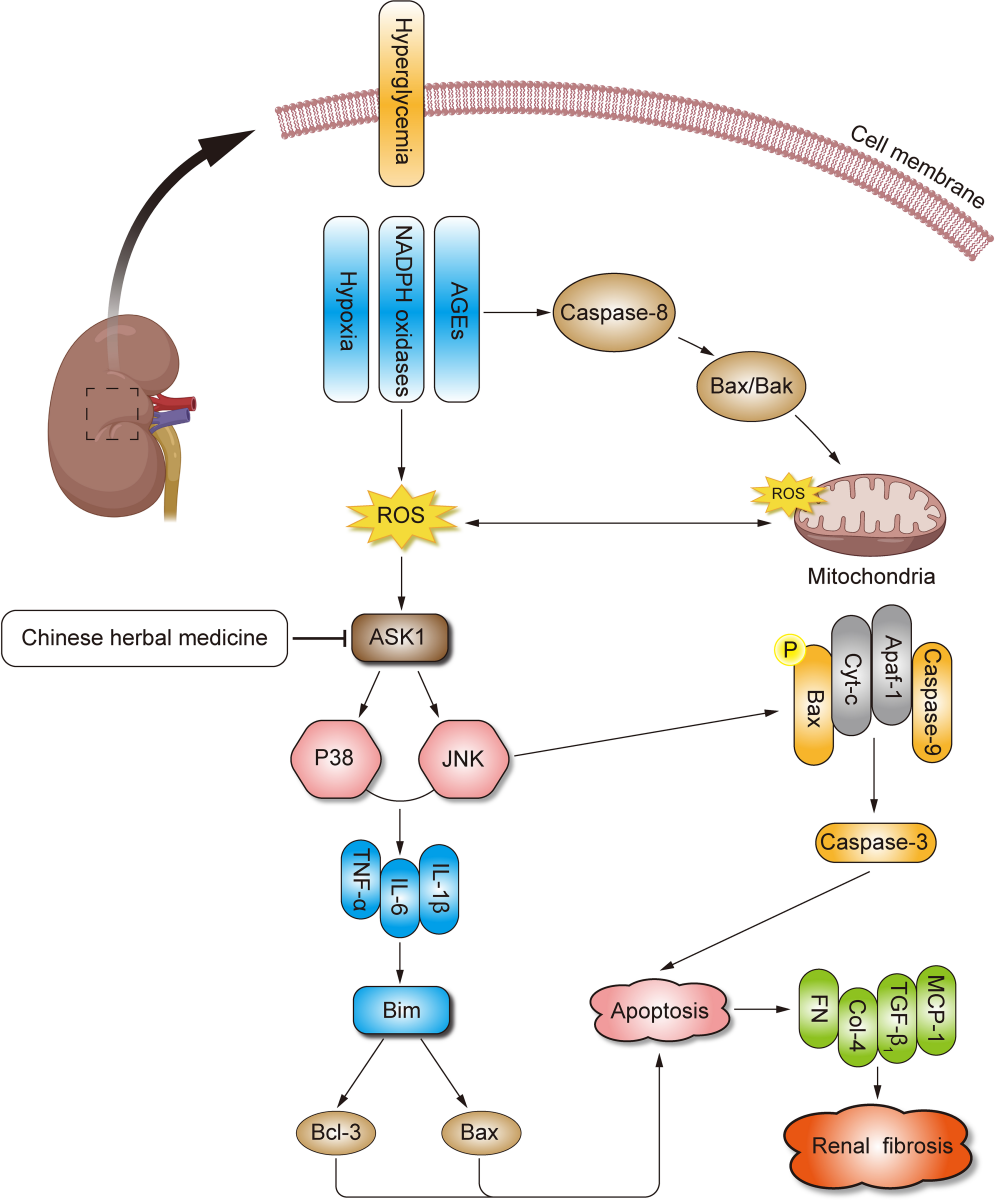
Mechanisms of CHM for DN. DN, diabetic nephropathy; CHM, Chinese herbal medicine; OS, oxidative stress; ECM, extracellular matrix; TGF-β, transforming growth factor-β; ROS, reactive oxygen species; AGEs, advanced glycation end products; MCP-1, monocyte chemoattractant protein-1; JNK, c-Jun N terminal kinase; AP-1, activator protein-1; ASK1, apoptosis signal-regulating kinase 1; MAPK, mitogen-activated protein kinase; NASH, non-alcoholic steatohepatitis; STZ, streptozotocin.

CHM has demonstrated anti-inflammation and antifibrotic efficacy in several studies in rodents with renal fibrosis. We will carry out studies using CHM as an ASK1 inhibitor in an animal model to retard the progression of renal fibrosis in DN. When available, the data from the established DN rodents will be critical to further clarify the beneficial therapeutic properties of CHM as a potential ASK1 inhibitor, making it an ideal candidate therapy for treating patients with DN (**Figure 1**).

## Author contributions

SW and SQ contributed to the conceptualization, writing and editing, and original draft preparation. BC contributed by analyzing the data and editing the table and figure. JZ and QC contributed through supervision, validation, reviewing, and funding. All authors contributed to the article and approved the submitted version.
